# Concurrent hypereosinophilic syndrome and deep vein thrombosis after Pfizer‐BioNTech COVID‐19 vaccination: A case report

**DOI:** 10.1002/ccr3.7001

**Published:** 2023-03-02

**Authors:** Shungo Yano, Taiju Miyagami, Takayuki Furusaka, Nagamasa Kano, Toshio Naito

**Affiliations:** ^1^ Department of General Medicine Juntendo University Faculty of Medicine Tokyo Japan

**Keywords:** adverse events following immunization, COVID‐19, case report, deep vein thrombosis, hypereosinophilic syndrome

## Abstract

Herein, we report a case of eosinophilia syndrome and deep vein thrombosis presenting concurrently after the administration of the BNT162b2 mRNA‐based coronavirus disease 2019 (COVID‐19) vaccine. It is extremely rare to have both hypereosinophilic syndrome and deep vein thrombosis simultaneously. Both are serious diseases and should be treated with caution.

## Abstract 2

We experienced a complicated case of eosinophilia syndrome and deep vein thrombosis after administration of BNT162b2 mRNA‐based coronavirus disease 2019 (COVID‐19) vaccine. It is extremely rare to have both hypereosinophilic syndrome and deep vein thrombosis simultaneously. Both are serious diseases and should be treated with caution.

## 1 CASE PRESENTATION

A 53‐year‐old Chinese man with a three‐year history of diabetes mellitus received a second dose of the BNT162b2 mRNA‐based COVID‐19 novel coronavirus vaccine (Pfizer‐BioNTech) 15 days prior to his visit to our hospital. He visited our hospital with a chief complaint of right thigh pain that had worsened 14 days prior to his visit.

The patient's blood glucose control was good with oral medication only. He had not used any new oral medications for the past several months and had no history of allergy.

**FIGURE 1:**
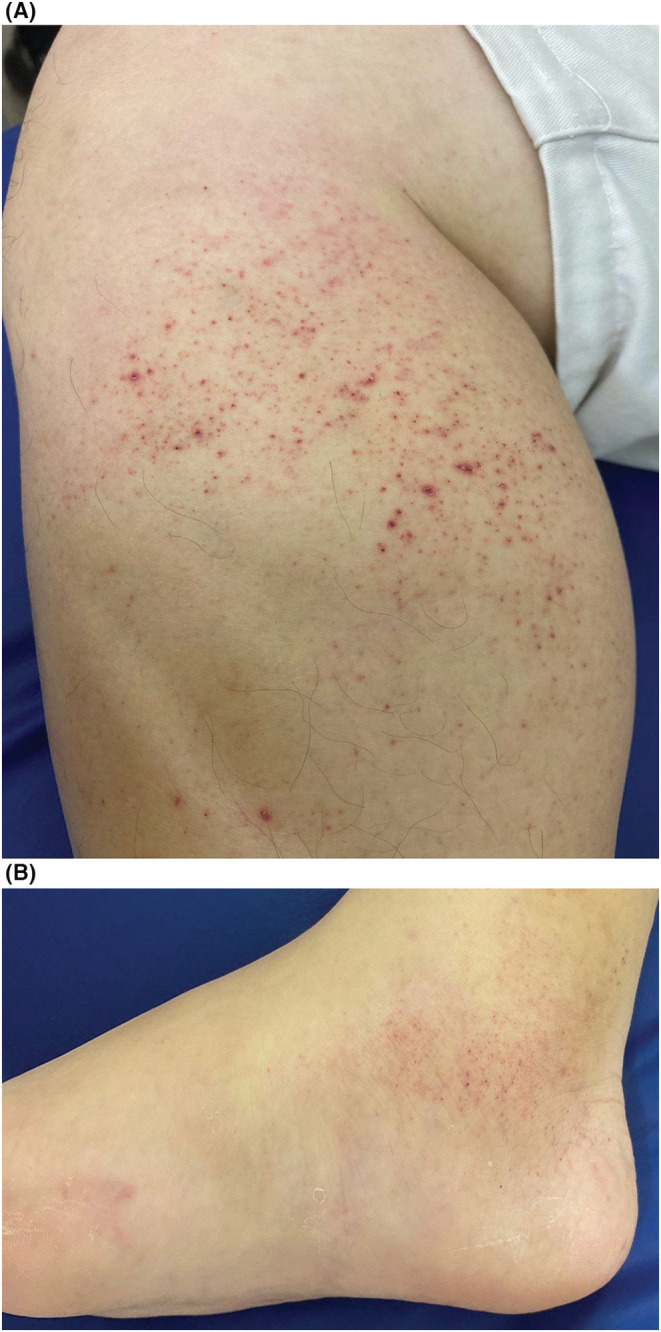
Imaging of the right lower leg (A) and the right ankle (B) in a man with painful purpura.

His consciousness was clear, and his vital parameters were as follows: body temperature, 36.4°C; blood pressure, 125/81 mmHg; heart rate, 88/min; respiratory rate, 14/min; and blood oxygen saturation (SpO_2_), 95% (on room air). Physical examination revealed no abnormal heart or respiratory sounds. Painful purpura were observed on the medial side below the right knee and on the medial side of the right ankle (Figure 1A,B).

Coldness and dysesthesia of the second and third fingers of the left hand and the first toe of the right foot were noted, with no other abnormal neurological findings. Laboratory tests showed leukocyte levels of 24,800/dL (reference range: 3900–9700), eosinophil levels of 14,880/dL (reference range: 70–440), and D‐dimer levels of 6.1 μg/mL (reference range: <1.0). Contrast‐enhanced computed tomography (CT) imaging from the neck to the lower leg showed a contrast‐impaired area from the right femoral vein to the popliteal vein. The patient had no predisposition to thrombogenicity and tested negative for antineutrophil cytoplasmic antibodies (ANCAs). Skin biopsy of the purpura showed eosinophilic infiltration of the subcutaneous and vessel walls.

Given a diagnosis of eosinophilic syndrome and deep vein thrombosis associated with COVID‐19 vaccination, the patient was started on prednisolone (1.0 mg/kg/day for 7 days) and rivaroxaban (15 mg twice/day). After 3 weeks, the dose was reduced to rivaroxaban only (20 mg/day). Treatment was completed in a total of 3 months. The patient's eosinophil count decreased promptly after prednisolone administration, and the swelling in the right lower leg reduced. After several months of follow‐up, the patient's neurological symptoms also improved and there were no additional hypereosinophilia flareups.

## 2 DISCUSSION

Deep vein thrombosis after vaccination with BNT162b2 mRNA is extremely rare.^1^ Moreover, eosinophilia has not been associated with deep vein thrombosis.

In general, venous thrombus is a symptom of organ damage in hypereosinophilic syndromes. However, it is very rare, accounting for 2.7% of all hypereosinophilic syndromes.^2^ Eosinophils have been reported to affect thrombus formation through their action as procoagulant phospholipids and through activating factor XII.^3^

Since both hypereosinophilic syndrome and DVT have a variable disease course and can rapidly worsen, and given the possibility of continuing routine vaccination against COVID‐19 in the future, monitoring and early diagnosis of these potentially severe vaccine complications is necessary.

## AUTHOR CONTRIBUTIONS

**Shungo Yano:** Conceptualization; data curation; methodology; project administration; visualization; writing – original draft; writing – review and editing. **Taiju Miyagami:** Conceptualization; data curation; formal analysis; methodology; project administration; resources; software; supervision; validation; visualization; writing – original draft; writing – review and editing. **Takayuki Furusaka:** Conceptualization; methodology; validation; writing – review and editing. **Nagamasa Kano:** Methodology; supervision; validation; writing – review and editing. **Toshio Naito:** Conceptualization; funding acquisition; investigation; validation; writing – review and editing.

## FUNDING INFORMATION

None.

## CONFLICT OF INTEREST STATEMENT

All authors have no pertinent conflict of interest to report for this manuscript.

## CONSENT

Written informed consent was obtained from the patient to publish this report in accordance with the journal's patient consent policy.

## Data Availability

No data was generated in reference to this case report.

## References

[1] Carli G , Nichele I , Ruggeri M , Barra S , Tosetto A . Deep vein thrombosis (DVT) occurring shortly after the second dose of mRNA SARS‐CoV‐2 vaccine. Intern Emerg Med. 2021;16:803‐804. doi:10.1007/s11739-021-02685-0 33687691 PMC7940863

[2] Ogbogu PU , Bochner BS , Butterfield JH , et al. Hypereosinophilic syndrome: a multicenter, retrospective analysis of clinical characteristics and response to therapy. J Allergy Clin Immunol. 2009;124:1319‐1325.e3. doi:10.1016/j.jaci.2009.09.022 19910029 PMC2829669

[3] Uderhardt S , Ackermann JA , Fillep T , et al. Enzymatic lipid oxidation by eosinophils propagates coagulation, hemostasis, and thrombotic disease. J Exp Med. 2017;214:2121‐2138. doi:10.1084/jem.20161070 28566277 PMC5502424

